# Impact of Estimation Uncertainty in PMU-Based Resynchronization of Continental Europe Synchronous Areas

**DOI:** 10.3390/s23052705

**Published:** 2023-03-01

**Authors:** Federica Costa, Lorenzo Peretto, Guglielmo Frigo

**Affiliations:** 1 Alma Mater Studiorum, University of Bologna, 40136 Bologna, Italy; 2 Swiss Federal Institute of Metrology METAS, 3003 Bern, Switzerland

**Keywords:** phasor measurement unit, frequency measurement, uncertainty analysis, resynchronization, transmission power system, dynamic signal model

## Abstract

Power system stability is a task that every system operator (SO) is required to achieve daily to ensure an uninterruptible power supply. Especially at the transmission level, for each SO it is of utmost importance to ensure proper exchange of information with other SOs, mainly in case of contingencies. However, in the last years, two major events led to the splitting of Continental Europe into two synchronous areas. These events were caused by anomalous conditions which involved in one case the fault of a transmission line and in the other a fire outage in proximity to high-voltage lines. This work analyzes these two events from the measurement point of view. In particular, we discuss the possible impact of estimation uncertainty on control decisions based on measurements of instantaneous frequency. For this purpose, we simulate five different configurations of phasor measurement units (PMUs), as characterized by different signal models, processing routines, and estimation accuracy in the presence of off-nominal or dynamic conditions. The objective is to establish the accuracy of the frequency estimates in transient conditions, more specifically during the resynchronization of the Continental Europe area. Based on this knowledge, it is possible to set more suitable conditions for resynchronization operations: the idea is to consider not only the frequency deviation between the two areas but also to take into account the respective measurement uncertainty. As confirmed by the analysis of the two real-world scenarios, such an approach would allow for minimizing the probability of adverse or even dangerous conditions such as dampened oscillations and inter-modulations.

## 1. Introduction

Modern power systems are characterized by an ever-increasing penetration of renewable energy sources and distributed generation. Such resources are typically interconnected via power converters that do not contribute to the overall system inertia [1,2]. Due to their inherent volatility, their contribution may vary rapidly and unpredictably. As a consequence, the power system is more prone to uncontrolled dynamics that may lead to dangerous transients or even blackouts, as proven by the recent events in Australia and Southern California [3,4].

In order to address these challenges, the monitoring and control infrastructure has to be suitably updated and improved [5]. In this sense, phasor measurement units (PMUs) represent a valuable solution. Based on their time stamps, it is possible to aggregate and compare measurements coming from different nodes and perform useful routines (e.g., state estimation and fault location) with an update rate of a few tens of ms [6,7].

It is worth noting that PMUs are not used only in a pre-contingency stage, but also in the gradual reconnection of loads and grid portions during system restoration [8,9]. In view of a successful reconnection, it is important that both the involved system areas have a comparable state, e.g., similar power signal parameters, in particular similar frequency. Indeed, frequency is an indicator of system stability as it accounts for the balance between power generation and consumption in the area.

By comparing the measurements of PMUs in the involved areas, it is possible to identify the most suitable time instant to perform an area reconnection. As proven by the recent literature, similar considerations also hold for load reconnection after under-frequency load shedding operations [10,11]. Each load reconnection has to be carried out with the system in quasi-stationary conditions, in order not to trigger uncontrolled oscillations or a new rapid frequency drop.

In this context, the recent literature has proposed several strategies to increase accuracy and minimize errors during specific situations. For instance, in [12], the authors aim at establishing the impact of the time synchronization deviation on synchrophasor measurements when the power system is subject to a dynamic state, whereas [13] presents three adaptive PMU models with wider linearity ranges than those specified in IEEE Std C37.118-1 [14]. Lastly, in [15], the authors present a model for magnitude compensation under frequency deviations for all the M-class filters to improve their performance. These works illustrate the impact of frequency deviations in different power systems’ conditions. However, to the best of the authors’ knowledge, there are no current works published in the literature which present the impact of the uncertainty of frequency measurements during the re-synchronization after a power system split. Moreover, for what concerns the regulatory framework, there are still some aspects concerning the measurements’ uncertainty that need to be tackled.

Indeed, in this scenario, the European Network of Transmission System Operators (ENTSO-E) has released a set of policies and operational guidelines. In particular, according to the Emergency Operations Policy (briefly, EOP), the frequency deviation between the two areas shall not exceed 200 mHz at the moment of the reconnection. This policy is quite conservative and guarantees a smooth and secure system operation [16]. However, this approach does not consider the uncertainty contributions inherent in any PMU-based measurement system.

Thanks to their large inertia, traditional transmission systems were sufficiently stable and easily controllable. In such a context, the response of the instrument transformer stage as well as the estimation errors of the PMU could be fully characterized a priori and suitably compensated [17,18]. It was thus reasonable to neglect the estimation uncertainty of the measurement infrastructure [19].

Conversely, modern transmission systems are characterized by reduced inertia and lower power quality (e.g., higher harmonic distortion and wide-band measurement noise) [20,21]. As a consequence, it is more difficult to properly characterize the behavior of the different components of the measurement chain. The instrument transformers may present a strongly non-linear response in the presence of spurious components [22,23]. PMUs, conceived for quasi-stationary conditions, may produce large errors in presence of fast dynamics [24,25].

Based on these considerations, this paper discusses the impact of PMU frequency estimation uncertainty in system restoration operations. More precisely, the paper does not intend to challenge the ENTSO-E policy or to propose an alternative strategy for a more stable and secure resynchronization. Conversely, this work aims at evaluating the actual impact of estimation uncertainty of PMU-based measurement of frequency in two real-world scenarios.

Since our target does not involve either the development of a new PMU model or the exploitation of simulated scenarios, we consider five well-known PMU models and two power system splits that occurred in recent years. As a matter of fact, for this analysis, we examine two cases of system split that took place in Croatia and France in 2021 [26,27]. Based on the official reports, we reproduce a numerically simulated version of the power signal, and we process them with PMU models representative of both M- and P-class of IEC/IEEE Std 60255-118-1 (briefly, PMU Std) [28]. In this context, we evaluate the PMU estimation errors and compare them with their expected uncertainty levels. Based on these results, we discuss the reliability of the PMU-based measurements and their comparability with the 200 mHz threshold set by ENTSO-E guidelines [16]. In this work, we will focus on the importance of assessing not only the frequency by means of PMUs during dynamic conditions but also its uncertainty. Indeed, it will be demonstrated how large frequency differences can lead to wide oscillations and longer transients that could affect machines and loads connected to the grid.

The paper is organized as follows. Section 2 describes the simulation model of the considered PMUs and the processing routine for the reconstruction of the power signal. In Section 3 and Section 4, we introduce the two test cases, referring to the system splits in Croatia and France, respectively, and we assess the PMUs’ performance in terms of estimation error and reliability. Section 5 discusses the obtained results and draws some useful conclusions about this work.

## 2. Simulation Models

In this section, we introduce the PMU simulation models and the processing routine to reproduce the power signals during the considered test cases. In this regard, it is worth noting that the resulting power signals do not consist of real-world acquisition and may be considered an oversimplification of the system behavior. In the absence of official waveform recordings, the best way to reproduce realistic power signals consists of their reconstruction based on the substations’ data disclosed in the official reports [26,27]. Nevertheless, despite being a reconstruction, these power signals can represent a plausible operating condition for PMUs installed in modern transmission systems.

### 2.1. PMU Models

For this analysis, we simulate five different PMU models, as taken from recent literature on synchrophasor estimation and representative of both P- and M-class of the PMU Std. In particular, we consider: the PMU Std reference algorithm for P-class [28], the compressive sensing Taylor–Fourier multifrequency (CS-TFM) model [29], and the iterative interpolated DFT (i-IpDFT) [30]. Being compliant with both classes, the last two algorithms have been simulated in P- and M-class configurations.

In principle, P-class PMUs are intended for protection applications due to their fast responsiveness, whereas M-class PMUs are more suitable for measurement applications thanks to their remarkable accuracy. Based on these considerations, it would be preferable to rely on P-class PMUs for controlling switchgears and conversely to adopt M-class PMUs for defining the most suitable reconnection time. However, in reality, not all substations are equipped with devices of both classes: a robust resynchronization protocol should also account for possible class inconsistencies between the PMUs in the two areas.

For the sake of simplicity, we refer to the different PMU models with the acronyms:

- PMU A: PMU Std reference algorithm, P-class configuration;
- PMU B: CS-TFM algorithm, P-class configuration;
- PMU C: i-IpDFT algorithm, P-class configuration;
- PMU D: CS-TFM algorithm, M-class configuration;
- PMU E: i-IpDFT algorithm, M-class configuration.

It is worth emphasizing that the goal of this work is not to develop or improve a specific PMU model. Rather, it aims to assess the performance of different PMU models that are compliant with the PMU Std under specific power system conditions. For the sake of clarity, in the following we provide a description of the five selected PMU models, highlighting the main advantages and disadvantages with a focus on frequency measurements.

**General settings:** In order to guarantee a unified simulation environment, we fix some parameters related to waveform acquisition and measurement reporting. This setting is applied to all the considered PMU models without loss of generality and is inspired by typical values in the synchrophasor estimation literature. The sampling and reporting rates are set equal to 12 kSa/s and 50 fps, respectively. Since both test cases occurred within the Continental European network, we assume a nominal system rate of 50 Hz. In compliance with the PMU Std latency requirements, the PMU models adopt an observation interval of three and five nominal cycles for P- and M-class, respectively.

**PMU A:** PMU A is derived from the PMU Std reference algorithm for P-class. In this sense, the PMU adopts a sliding window filtering approach. In line with the simulation general settings, the window length has been enlarged from two to three nominal cycles. This slight modification does not affect either the accuracy or the responsiveness of the model and guarantees a more rigorous and fair comparison of the measurement results.

The filter consists of a triangular-weighted finite impulse response filter. By suitably setting the filter parameters, it is possible to obtain a filter pass bandwidth centered around 50 Hz and covering the expected variation range of the fundamental frequency (namely, from 48 to 52 Hz). In order to maximize the PMU accuracy in off-nominal conditions, the phasor magnitude is corrected a posteriori based on the estimated frequency value.

PMU A relies on a static signal model, i.e., the acquired signal consists of a fundamental component in stationary conditions plus some narrow- and wideband distortions. Based on this assumption, the model state variables are essentially two: the magnitude and phase of the phasor associated with the fundamental component. Frequency and ROCOF are estimated as finite difference derivatives of the phase estimates. In particular, the frequency is computed taking into account two phase values, one before and one after the reporting time instant, i.e., it is always delayed by one reporting period with respect to the corresponding phasor. In a similar way, the ROCOF is computed as the second-order phase derivative, taking into account three phase angles relative to current, previous, and successive reporting periods.

PMU A advantages consist mainly of its reduced complexity which implies an easy implementation in any industrial controller. Moreover, the finite length of the filter response guarantees the minimization of the response time in the presence of transient conditions and the absence of oscillating trends as soon as the transient is out of the considered observation interval. On the other hand, PMU A relies on a stationary assumption that is hard to verify in modern power systems, even on short observation intervals. The peculiar differentiation strategy for the definition of frequency and ROCOF produce a low-pass filtering effect. As a consequence, the resulting estimates exhibit a smoother trend but are delayed with respect to the instantaneous value. Finally, this PMU model is severely affected by spurious injections from close-by spectral components (e.g., low-order harmonics and inter-harmonics).

**PMU B:** PMU B consists of an optimized formulation of the Taylor–Fourier Transform (TFT), as presented in [31]. With respect to the original formulation presented in [29], this version of the algorithm has been optimized to fit in a typical industrial controller setup, i.e., with an optimal trade-off between computational complexity and estimation accuracy.

The Taylor-Fourier Transform is an extension of the more traditional DFT [32]. By suitably modifying the transform kernel basis, it is possible to compute not only the phasor associated with a given frequency but also its Taylor expansion terms. In this case, the Taylor expansion is truncated at the second order. As a consequence, the model state variables are six: the phasor magnitude and phase plus their first- and second-order time derivative as computed in the reporting time instant.

In this sense, it is reasonable to say that the TFT-based approaches adopt a dynamic signal model. The inclusion of higher-order derivative terms produces significant advantages. In the presence of non-stationary conditions, the estimation accuracy is optimized: the higher-order phasors account for the parameter variations, whereas the zero-order phasor is the best approximation of the fundamental component at the reporting time instant. Moreover, the TFT can directly compute the instantaneous frequency and ROCOF, without any filtering effect or group delay introduced by finite differentiation operations.

On the other hand, the recent literature has proven that TFT performance is strongly dependent on the formulation of its kernel basis [33,34]. In particular, a prior yet coarse knowledge of the frequency of the most significant components (i.e., fundamental and distortions) is required. If a component is neglected or badly located, the TFT results might suffer from uncompensated spectral leakage and thus lead to inaccurate estimates, particularly on the higher-order derivative terms [35].

For this reason, the CS-TFM algorithm consists of three main stages. First, a CS-based routine identifies the signal spectral support, i.e., the frequencies of the most significant spectral components. Then, a TFM model is defined in order to reject all unwanted spectral interferences. Finally, the acquired observation interval is projected over the TFM kernel basis producing an estimate of the six state variables.

The recent literature has discussed several possible improvements for the CS-TFM, with a particular focus on the minimization of higher harmonic distortion [25]. Since the considered test cases refer to a transmission network scenario, it is reasonable to expect a reduced distortion level. Therefore, in the present implementation, we do not apply any windowing function to minimize the spectral leakage, and we consider a second-order expansion for the fundamental component, while we limit the other spectral components to the first order.

**PMU C:** PMU C is derived from the iterative interpolated DFT algorithm. In line with the simulation general settings, the parameters have been suitably modified for an observation interval of three nominal cycles.

The literature has widely discussed the possibility of interpolating DFT coefficients for the identification of sinusoidal component frequencies in non-synchronous sampling conditions [36]. However, this algorithm suffers from two types of spectral leakage. The long-range spectral leakage is due to the decaying lobes of the negative image component and becomes more and more relevant on short observation intervals. The short-range spectral leakage is caused by the interference of close-by components (i.e., harmonics and inter-harmonics).

The i-IpDFT algorithm adopts an iterative routine to minimize both long- and short-range spectral leakage. As shown in [30], the algorithm proves to be compliant with both P- and M-class requirements and is therefore an ideal candidate for this analysis.

As with any other DFT-based approach, the i-IpDFT relies on a static signal model. In particular, there are three model state variables, namely the magnitude, phase, and frequency of the fundamental component. The ROCOF, instead, has to be computed by means of a finite difference between two consecutive frequency estimates.

Given the static signal model, the i-IpDFT presents remarkable performance in quasi-stationary conditions, whereas it suffers from significant performance degradation in dynamic conditions. Indeed, the i-IpDFT provides the best stationary approximation of the signal captured in the observation interval. In the presence of parameter time variations, the DFT representation may produce erroneous or delayed and filtered estimates.

**PMU D and E:** The PMU D and E models consist of the M-class configuration of PMU B and C, respectively. The main difference is represented by the enlarged observation interval: from three to five nominal cycles, while the other parameters are kept unaltered. The longer observation interval results in a finer spectral resolution (from 16.6 to 10 Hz). Regarding the CS-TFM algorithm, the resolution enhancement allows for a more precise and stable definition of the signal spectral support and thus for a more effective rejection of spurious injections. Regarding the i-IpDFT algorithm, a finer resolution corresponds to a larger separation between the DFT bins associated with the fundamental and the non-informative components. In this way, the iterative routine is most likely to converge to its global optimum and minimize the estimation errors.

### 2.2. Simulated Power Signals

As further discussed in the following section, the test cases refer to reconnection operations between two system areas. In this context, we consider two power signals representative of each area. In the absence of waveform recorders, we reconstruct the power signal based on the official ENTSO-E reports [26,27].

For this analysis, we consider the voltage magnitude and frequency time profiles of two nodes close to the reconnection point. In more detail, the time profiles are interpolated via a non-linear fit routine using a shape-preserving piece-wise cubic polynomial. This allows for recovering a time domain power signal without discontinuities and is extremely consistent with the original profile. Moreover, given the analytical formulation of interpolated frequency, it is possible to retrieve the corresponding phase and ROCOF profiles by integration and derivation, respectively.

This method allows for defining the ground-truth values for phasor, frequency, and ROCOF at each time instant [37]. Evidently, this is not an exact reproduction of the real-world event and risks overfitting the estimation results presented in the original reports. Nonetheless, it presents similar features in terms of parameter values and spectral content. Based on the knowledge of ground-truth values, we can assess the actual PMU estimation errors and compare them with the expected uncertainty as given by the PMU Std.

In order to reproduce the uncertainty contributions of the PMU analog front-end (e.g., instrument transformer non-linearities, imprecise internal clock), the test waveforms are corrupted with an additive, uncorrelated noise with a signal-to-noise ratio (SNR) of 80 dB. Such a value of SNR has been chosen based on the fact that measurements performed at a transmission system level are generally characterized by lower levels of noise.

## 3. Test Case 1: Croatia—8 January 2021

This section illustrates the first test case that deals with the splitting of Continental Europe (CE) into two synchronous areas. This contingency occurred on 8 January 2021 in Ernestinovo, Croatia. In the following subsections, the system status before the contingency is detailed. This is followed by a description of the transient and sequence of faults. Lastly, a post-fault analysis is presented. More in detail, an error and uncertainty analysis is carried out on frequency estimates based on PMU measurements in the two CE areas.

### 3.1. Pre-Fault Power System Status

This subsection illustrates the power system status before the sequence of events that led to the splitting of the CE area into two separate regions.

On the day of the contingency, the power system was characterized by a high active power flow from the East to the West region. This situation was influenced by warm weather and Orthodox Christmas holidays in Southeast Europe, thus resulting in very low power demand, as well as a cold spell characterized by high demand in Northwest Europe. This condition led to an overall lower demand than usual in the Balkan Peninsula and a high power export from this area to CE of around 3900 MW.

In addition, the 110 kV overhead line connecting substation Majdanpek 1 to Majdanpek 2 was switched off due to a circuit breaker failure in Majdanpek 1. The power grid topology was not altered after the scheduled outage, counting on control room supervision of the power flows through the busbar coupler, protected by an over-current relay.

### 3.2. Contingency and Post-Fault Analysis

Based on the previous considerations, it can be concluded that the operation of the transmission system was on edge. As a matter of fact, a single outage was sufficient to drive the system to exceed the transient stability limit.

At 14:04:25.9 CET, the busbar coupler overload protection in Ernestinovo, Croatia led to a cascade of events. This implied the tripping of protections in other Croatian substations, in Serbia, Romania, and Bosnia Herzegovina.

After 15 s, the stabilization was already reached owing to the following countermeasure actions, both automatic and manual. Among these, there was the activation of frequency control response (FCR) in both the Northwest and Southeast areas and the automatic disconnection of 975 MW of generation and the automatic import of 447 MW of supportive power from the North synchronous area and 57 MW from GB. Additionally, 1.7 GW of interruptible services in France and Italy were disconnected.

**Figure 1:**
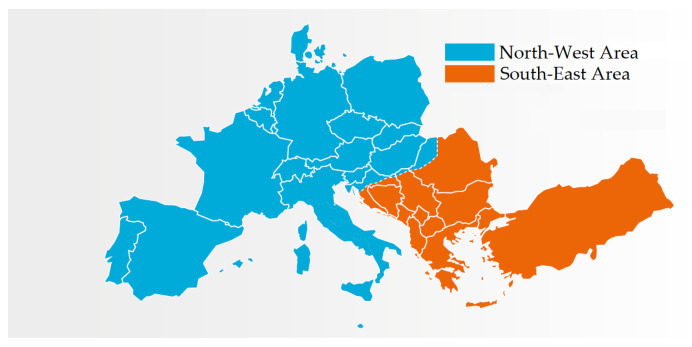
Test Case 1: Northwest and Southeast synchronous areas resulting from the system split on 8 January 2021 in blue and red, respectively. Adapted from [26].

Nevertheless, after the trip of a second element, i.e., the Subotica-Novi Sad transmission line at 14:04:48.9, the two areas started to separate from each other due to angular instability. The resulting split of the CE is shown in Figure 1.

**Figure 2:**
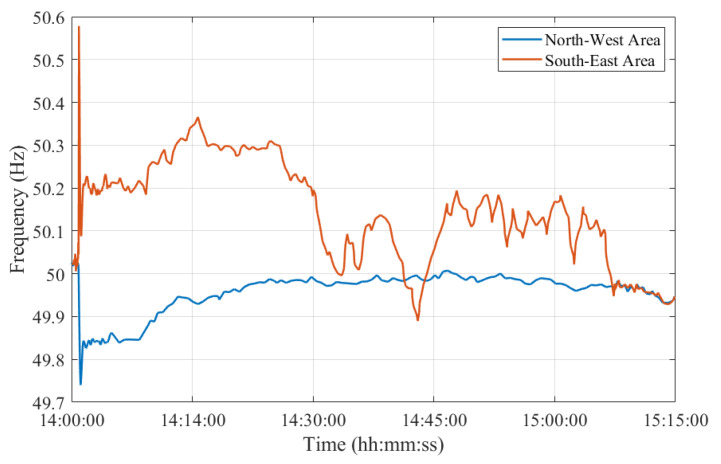
Test Case 1: Frequencies in the Northwest and Southeast synchronous areas measured during the entire contingency in blue and red, respectively. Adapted from [26].

The separation phenomena were characterized by a very fast voltage collapse at all substations close to the line of separation and by a gradual difference in the frequencies of the two areas. Indeed, the frequency was increasing in the Southeast area and decreasing in the Northwest area, as presented in Figure 2.

More specifically, frequency peaks of 50.6 Hz and 49.74 Hz were assessed in the Southeast and Northwest areas, respectively, as in Figure 2. ROCOF values reached up to 300 mHz/s and −60 mHz/s in the Southeast and Northwest areas, respectively.

### 3.3. PMU-Based Frequency Uncertainty Analysis

In this study, we consider one PMU per synchronous area. For what concerns the Northwest Area, we consider PMU measurements obtained at the substation located in Ernestinovo/Krsko, Croatia. For what concerns the Southeast Area, instead, we consider PMU measurements at the substation in Hamitabat, Turkey. For the sake of brevity, the first is referred to as NW, whereas the latter as SE hereinafter.

The objective is not only to establish a frequency estimate in the two areas, by means of PMUs having different dynamic performances but also to evaluate their errors in transient conditions. Based on this analysis, it would be possible to set a more robust and rigorous criterion for the resynchronization. More in detail, for this case study, we focus just on the resynchronization procedure which lasted from 15:00:00 CET until 15:15:00 CET. The reconnection of the SE Area to the CE was successfully accomplished at 15:07:31.6 CET.

The frequency error $f_{e}$ measured in Hz is computed as follows:

(1) $$f_{e}=f_{p}-f_{t}$$

where $f_{p}$, in Hz, represents the frequency measured by the PMU and $f_{t}$, in Hz, is the ground-truth frequency regarded as a reference and obtained from the interpolation.

**Figure 3:**
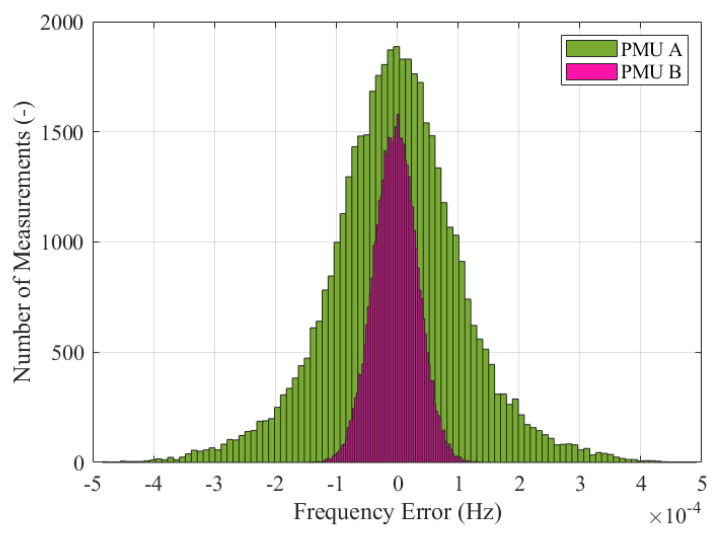
Test Case 1: Frequency error distribution for both PMU A (in pink) and B (in green), measured in the SE synchronous area during the resynchronization to CE.

As a first step, the method used to assess the accuracy of the frequency estimates involves the analysis of the error distributions shown in Figure 3. For the sake of readability, only the distributions of PMU A and B are displayed, but similar considerations hold for the other PMU models. The two histograms represent the statistical distribution of the frequency errors, $f_{e}$, computed using Equation (1) and produced by PMU A and B in pink and green, respectively. These results are obtained using the simulation and PMU model parameters illustrated in the previous Section 2 in the SE synchronous area during the re-synchronization to CE. The two histograms are characterized by quite different variation ranges. The worst-case error is limited to 0.1 and 0.4 mHz for PMU A and B, respectively. As expected, PMU A proves to be more accurate thanks to the higher noise rejection and improved dynamic tracking capability. Nonetheless, both PMUs are compliant with the PMU Std limit for off-nominal frequency conditions, namely 10 mHz. Applying the method in Figure 3 to all PMU models, it is possible to immediately derive some useful conclusions regarding the frequency errors’ variation ranges. The further analysis, instead, aims at correlating these results with the maximum frequency tolerance indicated in ENTSO-E EOP at the re-synchronization stage [16].

**Figure 4:**
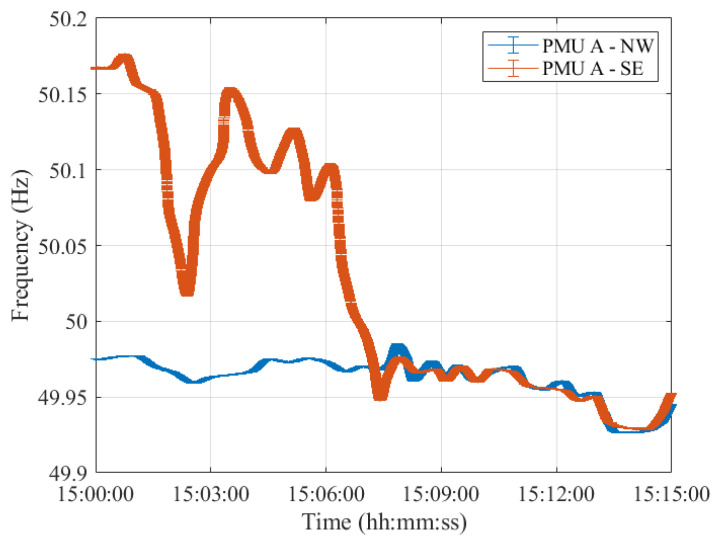
Test Case 1: Uncertainty on frequency estimates during the resynchronization process obtained using PMU A in the NW and SE synchronous areas in blue and red, respectively.

**Figure 5:**
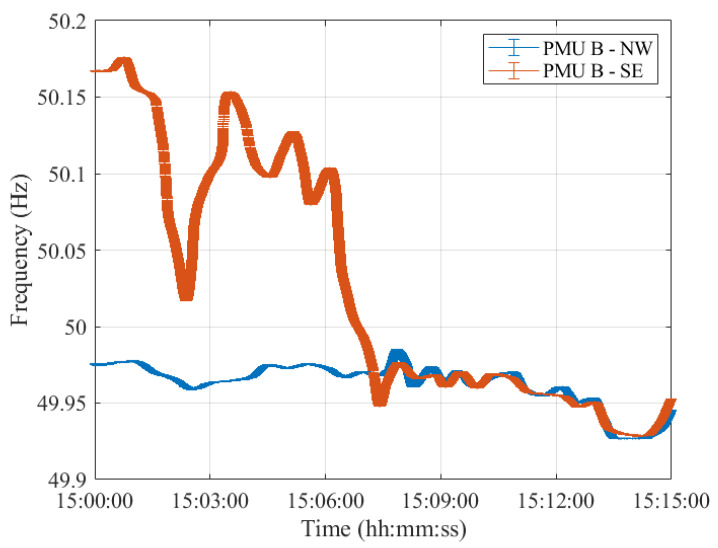
Test Case 1: Uncertainty on frequency estimates during the resynchronization process obtained using PMU B in the NW and SE synchronous areas in blue and red, respectively.

**Figure 6:**
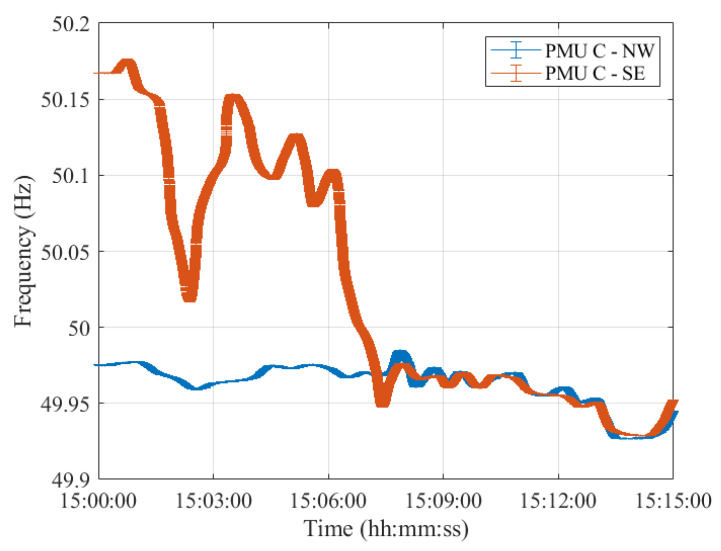
Test Case 1: Uncertainty on frequency estimates during the resynchronization process obtained using PMU C in the NW and SE synchronous areas in blue and red, respectively.

**Figure 7:**
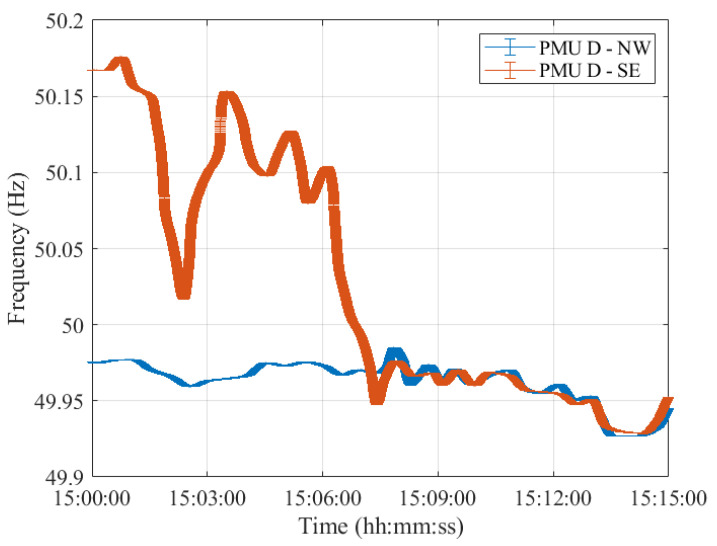
Test Case 1: Uncertainty on frequency estimates during the resynchronization process obtained using PMU D in the NW and SE synchronous areas in blue and red, respectively.

**Figure 8:**
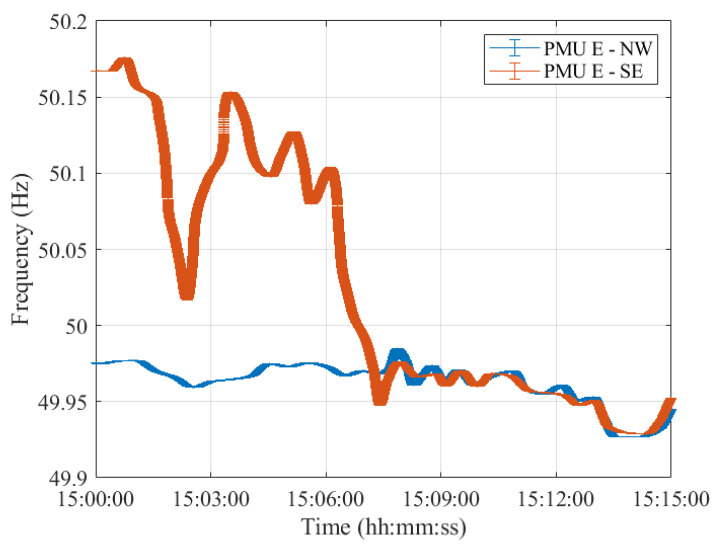
Test Case 1: Uncertainty on frequency estimates during the resynchronization process obtained using PMU E in the NW and SE synchronous areas in blue and red, respectively.

As a matter of fact, despite the different error variation ranges, the PMUs’ frequency profiles provided are comparable. In this regard, Figure 4, Figure 5, Figure 6, Figure 7 and Figure 8 show the measured frequency in correspondence with the reconnection operation: for each measurement, a vertical error bar indicates the corresponding error with respect to the ground-truth value as per Equation (1). This analysis is carried out for all five PMU models.

Although the resynchronization was successfully accomplished at 15:07:31.6 CET, it can be noted that at this time the frequencies and their corresponding uncertainty bandwidths in the two synchronous areas are not perfectly overlapping. However, this is the first time instant during which the two frequencies intersect. They are within the maximum tolerance of ±200 mHz to 50 Hz, indicated in ENTSO-E EOP [16]. Indeed, they fall within a maximum difference of 50 mHz. It is worth recalling that in this policy, approved on 26 September 2017, ENTSO-E suggests that *both systems must be in a stable state, and both frequencies must be near 50 Hz, with a maximum tolerance of ±200 mHz to 50 Hz, to resynchronize as securely as possible*. Based on this rationale, we considered such a threshold and compared the PMU frequency uncertainties with that. The same reasoning holds true for Test Case 2 in Section 4.

Moreover, it can be interesting to compare the frequency uncertainty bandwidths with the maximum frequency errors provided in the PMU Std. From the resynchronization time instant onwards, the two frequencies, jointly with their uncertainties, perfectly overlap, guaranteeing a correct power system synchronization.

It is interesting to observe how both PMU results align almost perfectly with those presented in [26]. It is thus reasonable to say that this test case is characterized by dynamics that can be easily captured by any P-class PMU. The use of M-class PMUs does not produce relevant changes in the estimates but may be considered if higher distortions are expected.

From this incident, ENTSO-E derived two main recommendations. Firstly, the substation topology should be chosen in such a way that the power flow through the busbar coupler is as low as possible. Secondly, it should be mandatory to include outages of any transmission elements in the contingency lists, including busbar couplers.

Furthermore, for what concerns the uncertainty on the estimated frequency, it can be concluded that both PMU models are able to correctly assess the frequency in dynamic conditions, albeit better performances in noise rejection are obtained for PMU A. Nonetheless, both PMUs show errors within the maximum ones indicated in the PMU Std and within the ENTSO-E EOP [16]. Hence, these conditions ensured a correct restoration of the CE synchronous area without any frequency oscillations in either of the two areas.

## 4. Test Case 2: France—24 July 2021

This section illustrates the second test case that also deals with the splitting of CE into two synchronous areas. This contingency occurred on 24 July 2021 in Moux, France. In the following subsections, the system status before the contingency is detailed. This is followed by a description of the transient, the sequence of faults, and a detailed description of the resynchronization procedure which led to wide frequency oscillations. Lastly, a post-fault analysis is presented to include the uncertainty analysis on frequency estimates based on PMU measurements.

### 4.1. Pre-Fault Power System Status

This subsection presents the power system status before the sequence of events that led to the splitting of the CE area into two separate regions. On the day of the contingency, a fire broke out in the Moux area, in the south of France at approximately 13:30:00 CET. During the organization of the firefighting efforts, the fire department acknowledged that two 400 kV lines connecting Baixas–Gaudière were located in the fire area. However, these lines remained energized despite a request to the French TSO (RTE) to switch them off. In addition, there was a rather high power flow of 2544 MW from France to Spain.

### 4.2. Contingency and Post-Fault Analysis

The sequence of events that led to the splitting of the CE into two areas started with the tripping of differential protection caused by a two-phase fault at a substation in Baixas, France at 16:33:12.0 CET.

The frequency, voltage, and load of the transmission elements remained within normal values, as expected after an N-1. However, the N-1 criterion was no longer fulfilled after this event, which is why the Spanish TSO (REE) and RTE agreed to reduce the exchange between France and Spain. Nevertheless, the next two trips occurred before this reduction became effective.

The second event occurred at 16:35:23.8 CET with the trip of 400 kV Baixas–Gaudière line 1. After this second line trip, the voltage started collapsing: a voltage degradation was visible and its phase angle difference started increasing. The coils started to be disconnected, and the first generation was lost.

The third event occurred at 16:36:37.0 CET, initiated by the trip of the Argia–Cantegrit line. This tripping caused the loss of synchronism between France and the Iberian Peninsula, after which, the only possible defense action was to split the system at already planned locations. However, the frequency in the Iberian Peninsula started to drop even before the three remaining interconnection lines between Spain and France had tripped.

**Figure 9:**
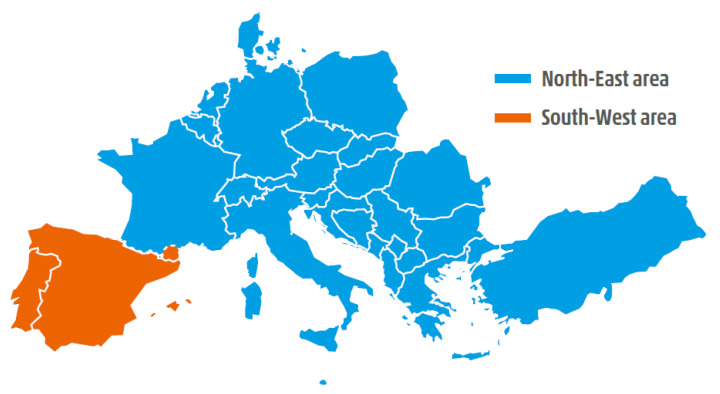
Test Case 2: Northeast and Southwest synchronous areas resulting from the system split on 24 July 2021 in blue and red, respectively. Adapted from [27].

There was a total load shed of 4872 MW of which 3561 MW was from REE. Due to early voltage issues, the coils (capacitors) start to be disconnected (connected) already before pump/load shedding (−1440 MVAr). The resulting split of the CE is shown in Figure 9.

More specifically, the nadir frequency measured in the middle of the Iberian Peninsula was 48.681 Hz, whereas, the maximum local ROCOF was measured at the Hernani substation in Spain and it was equal to −1.03 Hz/s.

### 4.3. PMU-Based Frequency Uncertainty Analysis

Similar to the analysis in the previous section, in this scenario, we consider one PMU per synchronous area. For what concerns the Northeast Area, we analyze PMU measurements obtained at the substation located in Saucats, France. Regarding the Southwest Area, instead, we consider PMU measurements at the substation in LaCereal, Spain. For the sake of brevity, the first is referred to as NE, whereas the latter as SW hereinafter.

With respect to Test Case 1 shown in Section 3, in this scenario, not only do we want to establish a frequency estimate in the two areas, but also to evaluate their errors in two transient conditions, i.e., during the fault sequence, and the resynchronization.

More in detail, we analyze the second and third faults leading to the splitting of the CE area, the time interval lasting from 16:35:00 CET until 16:42:00 CET. Then, we assess the PMU dynamic responses during the resynchronization procedure which lasted from 17:09:00 CET until 17:09:30 CET. The reconnection of the SE area to the CE was successfully accomplished at 17:09:00 CET.

**Figure 10:**
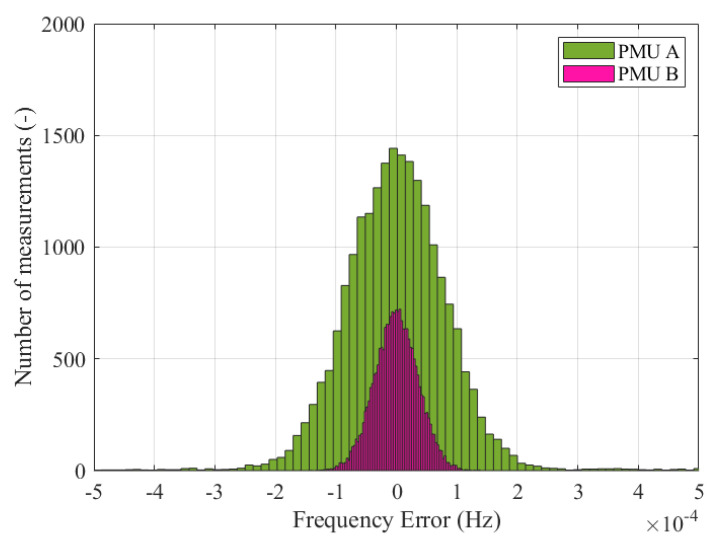
Test Case 2: Frequency error distribution for both PMU A (in pink) and B (in green), measured in NE synchronous area during the fault sequence leading to the splitting of CE.

Similar to Test Case 1, as a first step, we assess the accuracy of the frequency estimates by investigating the two error distributions shown in Figure 10. The two histograms refer to the measured frequency errors in the NE synchronous area during the fault sequence leading to the splitting of CE. For the sake of completeness, it is worth recalling that the two histograms represent the statistical distribution of the frequency errors, $f_{e}$, obtained applying Equation (1), produced by PMU A and B in pink and green, respectively. These results were obtained using the simulation and PMU model parameters illustrated in the previous Section 2, which are the same as for Test Case 1 described in Section 3.

Even in this case, PMU A and B present quite different variation ranges despite their mean values being the same. Namely, PMU B has worse dynamic performance with a worst-case frequency error of 0.5 mHz. whereas PMU A presents the same distribution width as in the previous case, i.e., spanning around ±0.1 mHz, indicating the robustness of the CS-TFM approach.

**Figure 11:**
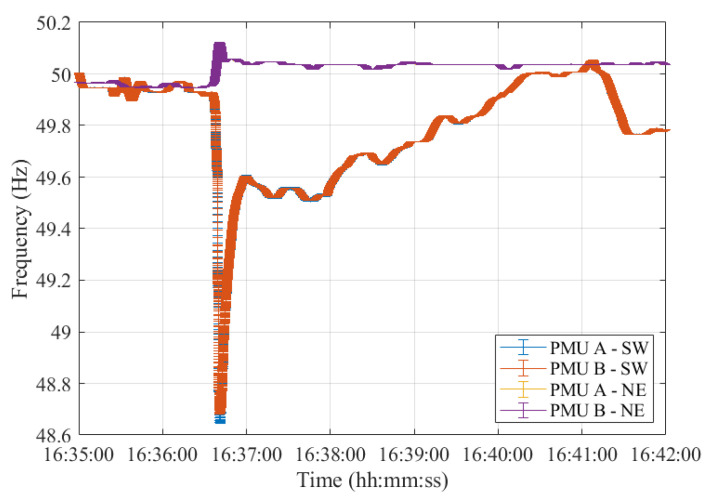
Test Case 2: Uncertainty on frequency estimates obtained using PMU A and B during the 2nd and 3rd faults, leading to the system separation in both SW and NE synchronous areas.

Figure 11 illustrates the error in the frequency estimates during the second and third events that resulted in the splitting of the CE. Both PMU models and synchronous areas are considered. These results align quite well with those presented in [27]: the frequency nadir of 48.68 Hz is correctly detected by both PMUs. Despite the overlapping of the results of PMU A and B, it is worth recalling that the latter presents a much wider error bandwidth in the frequency estimation.

In Figure 11, the time instant at which the second event occurs can be clearly noted. Indeed, at this time, the two frequencies oscillate around their rated value of 50 Hz. Despite their error bandwidth overlapping for several seconds, this is not a suitable time instant to perform the resynchronization. Indeed, a few seconds after, at 16:36:37 CET, the third event occurred. It led to the splitting of the CE into two separate areas which are also indicated by the non-overlapping frequency estimates at this specific time instant. As a matter of fact, from this time onwards, the two frequencies diverge in opposite directions and never intersect for the following minutes.

**Figure 12:**
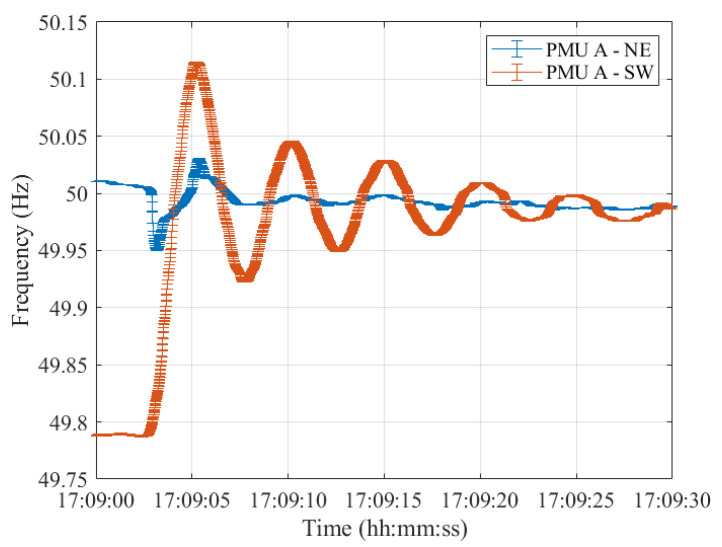
Test Case 2: Uncertainty on frequency estimates during the resynchronization process obtained using PMU A in the NE and SW synchronous areas in blue and red, respectively.

**Figure 13:**
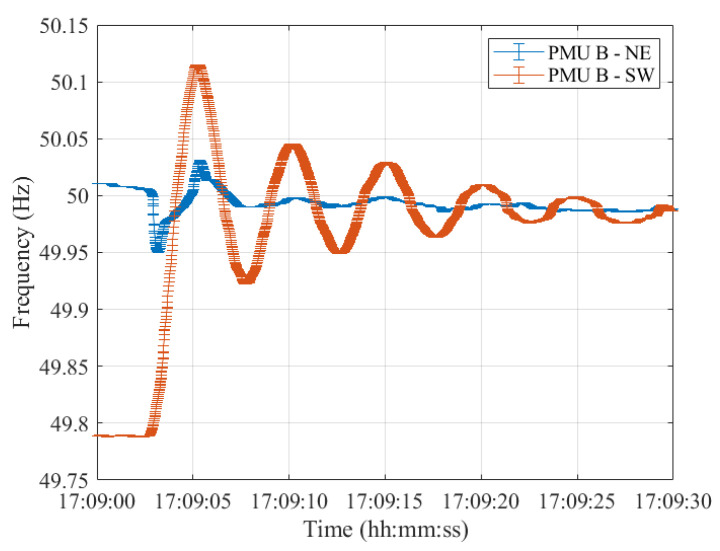
Test Case 2: Uncertainty on frequency estimates during the resynchronization process obtained using PMU B in the NE and SW synchronous areas in blue and red, respectively.

**Figure 14:**
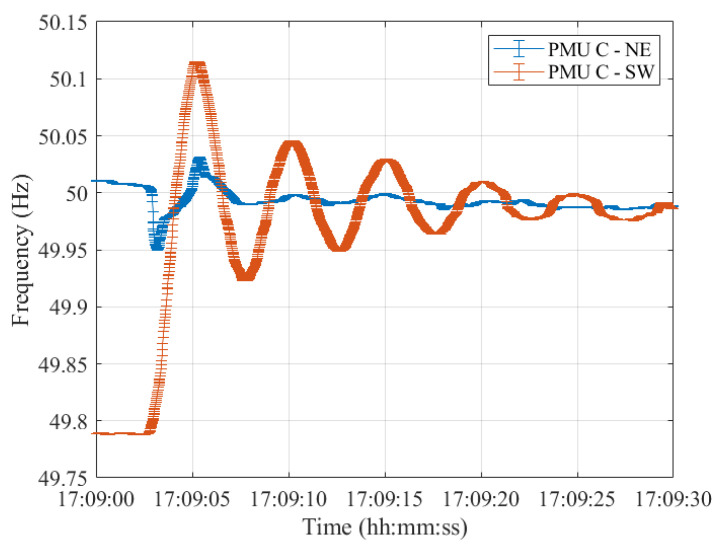
Test Case 2: Uncertainty on frequency estimates during the resynchronization process obtained using PMU C in the NE and SW synchronous areas in blue and red, respectively.

**Figure 15:**
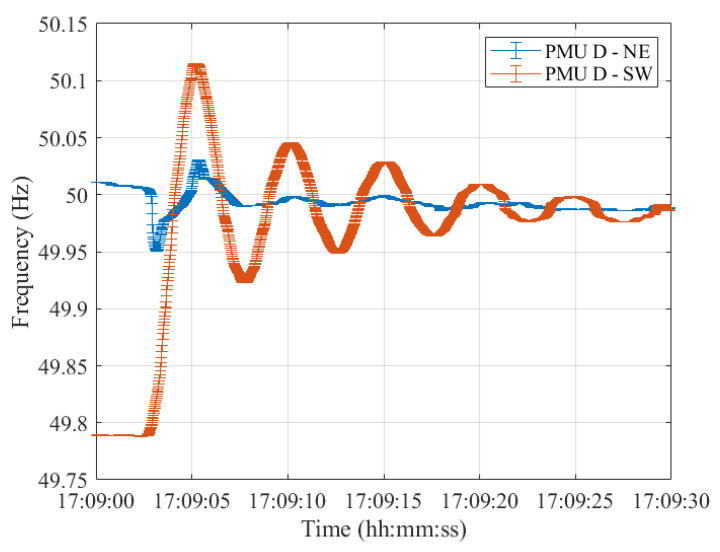
Test Case 2: Uncertainty on frequency estimates during the resynchronization process obtained using PMU D in the NE and SW synchronous areas in blue and red, respectively.

**Figure 16:**
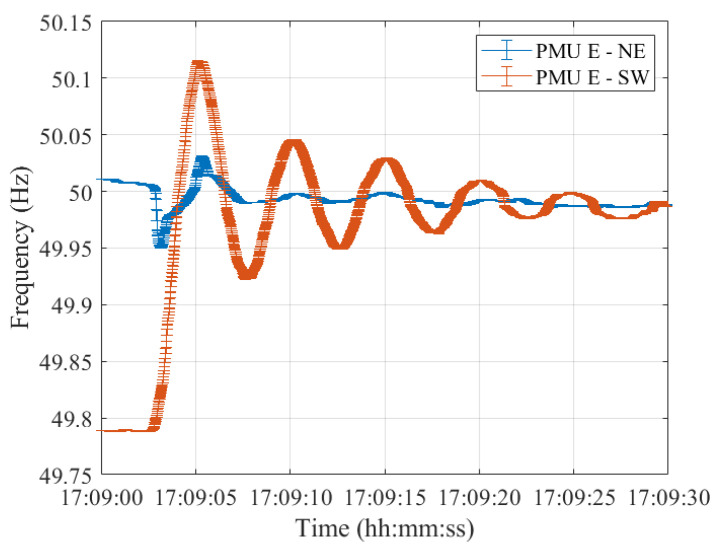
Test Case 2: Uncertainty on frequency estimates during the resynchronization process obtained using PMU E in the NE and SW synchronous areas in blue and red, respectively.

Lastly, resynchronization occurred at 17:09:00 CET. The frequencies measured in the two synchronous areas by the five PMU models are shown in Figure 12, Figure 13, Figure 14, Figure 15 and Figure 16, illustrating 30 s after the resynchronization. It can be noted how at the beginning of the procedure, the two frequencies, comprising their error bandwidths, are approximately within the ±200 mHz suggested by the ENTSO-E EOP [16]. Nevertheless, after the successful resynchronization, the two frequencies show a behavior that is clearly in contrast to what has been assessed in Test Case 1. In fact, the two frequencies do not overlap as in the previous scenario, but rather the frequency in the separated area, i.e., SW, starts oscillating around its rated value of 50 Hz. Just at the end of the transient, after 30 s, it can be concluded that the two frequencies are superimposing each other.

From this very first comment, one of the potential implications of our findings on the reliability of the power systems can be highlighted. As a matter of fact, it is not only the frequency that should be considered when performing power system maneuvers. It is evident how its uncertainty will be taken into account as it holds important information that should not be neglected, especially before performing emergency maneuvers or counteractions.

From this second incident, ENTSO-E derived several recommendations. Among these, they suggested supplementing important transit corridors with special protection scheme (SPS) functionality, in combination with automatic overload protection. First, the overload protection with a 1 to 5 min threshold will be complemented with SPS functionality, e.g., based on a centralized industrial load shedding scheme. Second, they propose the coordination of protection against loss of synchronism (DRS) with the protection schemes of neighboring systems. Additionally, ENTSO-E advised improving the communication chain in case of external conditions impacting system operation.

Furthermore, from the measurement point of view, we can derive that it is essential to evaluate the uncertainty to be associated with the frequency estimates. This is relevant, most importantly, during transient conditions since measurement devices, PMUs in our case, are prone to higher errors. Therefore, their outcomes can be strongly influenced. In addition, this case study showed the results of performing reconnection when the frequencies in the two areas are far apart. Indeed, when roughly 200 mHz can be assessed, this may result in strong frequency oscillations in the reconnecting area. Not only does this strain the mechanical parts of the synchronous machines but it also affects the loads which are not supplied at their rated frequency.

## 5. Results Discussion

This paper presented a study on the evaluation of the uncertainty of frequency estimates in the case of resynchronization of the CE synchronous areas.

To this purpose, we considered two different PMU models having distinct performances, especially in dynamic conditions. We examined a PMU relying on a dynamic signal model and a second one based on the P-class reference algorithm.

We reconstructed the time-varying signals by means of a non-linear fitting algorithm, and we considered the resulting values as the true values. A frequency error bandwidth was computed in transient conditions, i.e., during the sequence of faults and the resynchronization procedure, for both PMUs. The results indicate how having a dynamic signal-model-based PMU ensures better tracking of transients and noise rejection.

Two test cases were taken into account. More in detail, the first one refers to the Croatia contingency, and the second one to the French one. These two scenarios are both characterized by a sequence of events that led to the splitting of the CE area into two independent synchronous regions. However, in the first case, resynchronization was successfully achieved. while in the second scenario, it was performed when the two frequencies were still too far apart from each other, resulting in wide oscillations.

By analyzing the frequency estimates jointly with their error bandwidth it is possible to observe how associating an uncertainty to a frequency result is of utmost importance, especially during transient conditions.

In fact, the first case study showed a perfect overlap between the frequency error bandwidths in the two synchronous areas after the resynchronization. In contrast, in the second case, the frequency of the area to be reconnected started oscillating around its rated value since the two frequencies were barely within ENTSO-E guidelines.

Indeed, recalling ENTSO-E’s EOP guideline, it is interesting to notice that the ±200 mHz suggested by the policy could be insufficient to ensure a resynchronization as secure as possible. As proven by the results obtained in this study, comparing this threshold with the PMU frequency uncertainties, it is possible to conclude that the two frequencies should have overlapping error bandwidth in order to guarantee a safe resynchronization.

Therefore, the results of this research indicate the relevance of the estimation of the uncertainty to be associated with time-varying quantities before and after performing counteractions on power systems. Additionally, by means of PMUs, it is feasible to obtain time-stamped measurements that can be provided at high reporting rates, ensuring prompt response in case of power system events.

## Data Availability

The data are available upon request to the corresponding author.
